# Neonatal Outcomes after Combined Opioid and Nicotine Exposure in Utero: A Scoping Review

**DOI:** 10.3390/ijerph181910215

**Published:** 2021-09-28

**Authors:** Krystyna R. Isaacs, Sravanthi Atreyapurapu, Amal H. Alyusuf, David M. Ledgerwood, Loretta P. Finnegan, Katie H. K. Chang, Tony X. Ma, Yukiko Washio

**Affiliations:** 1 Benten Technologies, 9408 Grant Ave Suite 206, Manassas, VA 20110, USA; krysisaacs@gmail.com (K.R.I.); sravaa@yahoo.com (S.A.); aalyusuf@bententech.com (A.H.A.); hkchang@bententech.com (K.H.K.C.); tonyma@bententech.com (T.X.M.); 2 Department of Psychiatry and Behavioral Neurosciences, Wayne State University School of Medicine, 3901 Chrysler Service Drive, Detroit, MI 48201, USA; dledgerw@med.wayne.edu; 3 Executive Officer of the College on Problems of Drug Dependence and Finnegan Consulting, Philadelphia, PA 19140, USA; finnegal337@gmail.com; 4 Substance Use, Gender and Applied Research, RTI International, 3040 E Cornwallis Rd, Durham, NC 27709, USA; 5 Department of Obstetrics, Gynecology and Reproductive Sciences, Temple University Lewis Katz School of Medicine, 3500 N Broad St, Philadelphia, PA 19140, USA

**Keywords:** tobacco exposure, opioid use disorder, pregnancy, neonatal abstinence syndrome, nicotine

## Abstract

Background: The majority of women who are pregnant with opioid use disorder (OUD) also smoke tobacco but are rarely offered tobacco cessation counseling. While the effects of exposure to opioids and nicotine in utero are well-understood separately, understanding the impact of the combined exposure to these substances on neonatal outcomes is lacking. Methods: A scoping review was conducted using PubMed and Scopus databases for studies addressing the combined exposure to opioids and nicotine during pregnancy published between 1 January 1980 and 9 July 2019. A total of 29 papers met the eligibility criteria for inclusion, with nine being identified as clinical trials (three from the MOTHER study) and two as secondary data analysis of clinical trial data. Results: Neonatal outcomes for infants who had a combined exposure to opioids and nicotine in utero indicated a reduction in birth weight and birth length. Findings in infants exposed to both nicotine and opioids were mixed with regard to the duration of neonatal abstinence syndrome (NAS), the likelihood of treatment for NAS, doses of medicine used to treat NAS, and NAS scores when compared with infants who had opioid exposure without nicotine. Conclusions: The combined exposure to nicotine and opioids during pregnancy may lead to a reduction in neonatal birth weight and birth length and more severe NAS signs, compared with opioid use alone, but more research is necessary to identify the minimum dosage and length of nicotine exposure to accurately predict these outcomes.

## 1. Introduction

A significant increase in prenatal opioid use has been observed in recent years [1]. In 2016, 6% of women who are pregnant in the U.S. reported past-month illicit drug use, with 1.2% reporting opioid misuse [2]. Infants delivered to women who are pregnant with opioid use disorder (OUD) present with neonatal abstinence syndrome (NAS), a constellation of signs indicating central nervous system and gastrointestinal dysfunction, respiratory distress, and signs relating to the autonomic nervous system at a rate ranging from 50–80% [3,4,5,6].

Prenatal substance use (use of illicit substances, alcohol, and tobacco) is a modifiable risk factor for significant gestational complications for both women and their newborns [7,8,9,10]. Polysubstance use further increases the risk for adverse pregnancy outcomes, including miscarriage, low birth weight, preterm birth, stillbirth, and neurocognitive disorders [7,8,9,11,12]. Self-reported maternal smoking, alcohol use, and drug use each increase the likelihood of having neonatal morbidity, including impaired Apgar scores (<7 at 5 min postpartum), neonatal resuscitation, and neonatal intensive care unit (NICU) admission [13].

Between 80% and 90% of women who are pregnant and dependent on opioids also smoke cigarettes [4,14,15]. Although legal, cigarette smoking during pregnancy can lead to long-term adverse outcomes in offspring, independent of other illicit drug use [7,9,16]. Self-reported cigarette smoking (more than two cigarettes a day) exacerbates the likelihood of preterm birth by more than four times [11]. Prenatal tobacco use has also been reported to lead to adverse birth outcomes such as low birth weight and to exacerbate NAS severity [3,9,11]. The rate of achieving nicotine abstinence during pregnancy is poor (i.e., ~30%), relative to abstaining from other illicit and licit (e.g., alcohol) substances during pregnancy (i.e., >70%) [17]. As of 2016, only 48.9% of mental health treatment facilities and 64% of substance abuse treatment facilities screen for tobacco use, and few of these offer tobacco cessation counseling [18].

Methadone and buprenorphine can be used in medication-assisted treatment in women who are pregnant with OUD. Methadone is a long-acting opioid agonist and buprenorphine is a partial opioid agonist. Both are used to reduce cravings and withdrawal symptoms and are recommended to promote recovery [19,20]. Given that significant tobacco consumption in women who are pregnant and not taking opioids can result in infant nicotine withdrawal signs [21,22] and noticeable poor neonatal outcomes [9,23,24,25], it is critical to review whether prenatal exposure to both nicotine and opioids (for treatment of OUD, treatment of pain, or illicit use) has an additive effect. The current scoping review focuses on neonatal outcomes in infants with prenatal exposure to opioid agonist treatments, prescription opioids, or illicit opioids combined with tobacco.

This review will address the specific question “What is the impact of combined in utero exposure to opioids and nicotine on NAS and other neonatal outcomes?” The goal is to provide clinicians with a better understanding and inform public health efforts to develop appropriate interventions to support women who are pregnant with OUD in their attempts to either quit or reduce their nicotine consumption during pregnancy.

## 2. Methods

A scoping review of the literature for studies examining the impact of in utero exposure to both opioids and tobacco on neonatal outcomes was conducted utilizing a multi-step approach based on the Preferred Reporting Items for Systematic Reviews and Meta-Analyses (PRISMA) guidelines [26].

***Search Strategy***—PubMed and SCOPUS databases were searched by two independent authors for peer-reviewed English-language literature published between January 1980 and 9 July 2019. The Scopus database is the most comprehensive database of peer-reviewed literature from medicine and social sciences and includes over 76 million records sourced from over 39,000 series titles [27]. The PubMed search revealed no additional articles not already identified in the Scopus search. The search terms included Medical Subject Headings (MeSH) and non-MeSH terms including “Neonatal Abstinence Syndrome (NAS)”, “Neonatal Withdrawal Syndrome (NWS)”, “Neonatal Opioid Withdrawal Syndrome (NOWS)”, “Newborn Opioid Withdrawal Syndrome”, “Opioid NAS”, “Finnegan NAS”, “Opioid and Tobacco Exposure NAS”, “Tobacco Exposure Neonatal Abstinence Syndrome”, “Maternal Tobacco Exposure Neonatal Abstinence Syndrome”, “Opioid and Nicotine Exposure NAS”, “Nicotine Exposure Neonatal Abstinence Syndrome”, “Maternal Nicotine Exposure Neonatal Abstinence Syndrome”, and “Intervention Neonatal Abstinence Syndrome”.

***Study Eligibility Criteria***—The study inclusion criteria were set a priori before any searches began. The inclusion criteria were that peer-reviewed publications must (1) document NAS after in utero exposure to opioids and nicotine (e.g., alternate names for NAS such as NOWS or NWS are acceptable); (2) report a measurable outcome related to nicotine; and (3) include mothers who have had prior exposure to opioids and nicotine. In addition, only studies published in the English language and that followed the requirements for human subject research were included.

Studies were excluded if they were not related to maternal tobacco exposure; did not have measurable outcomes related to nicotine; were not published in a peer-reviewed journal; were not published in English; did not include humans as the subject of research; did not include maternal opioid exposures; or were not a primary source intervention report (i.e., reviews, case reports, surveys, etc.). Any discrepancies or concerns between the study raters were resolved in discussion with members of the research team (K.I., S.A., Y.W., and K.C.).

***Study Records***—Reference management and duplicate record elimination were conducted using RefWorks (copyrighted by ProQuest LLC, Ann Arbor, MI, USA). Testing the “exact duplicates” and “close duplicates” options occurred in RefWorks. “Exact duplicates” identified exactly matching references while “close duplicates” identified approximate matches with 95% accuracy. To avoid eliminating non-duplicates during the duplication-eliminating process, the elimination of “close duplicates” was conducted manually using Zotero software (George Mason University, Fairfax, VA, USA).

***Screening***—References were screened based on the relevance of the title and abstract. When the title contained key phrases of terms that indicated it may be pertinent to the effects of opioids with tobacco on NAS or neonatal outcomes, a thorough review of the abstract was completed. If the abstract was a probable match, based on our inclusion and exclusion criteria, the research team conducted a full-text review. The authors searched the bibliographies of articles selected for full-text review and added additional papers to be included in the full-text review.

***Data Items***—The PICO framework (i.e., Problem/Patient/Population, Intervention/Indicator, Comparison, Outcome [28]) was used to guide the determination of the effects of combined nicotine and opioid exposure on neonatal outcomes.

***Abstraction Table***—All pertinent data were collected from the articles and organized in an abstraction table with the following data elements:

- Publication information (authors, publication date, journal in which the study was published);
- Known substances of in utero exposure (i.e., tobacco, antidepressants, selective serotonin reuptake inhibitors (SSRIs), antipsychotics, etc.) identified through self-report, urinalysis, or meconium testing;
- Outcomes that were measured (i.e., birth weight, birth length, length of stay, etc.);
- Study design (i.e., retrospective cohort study, clinical trial, etc.);
- Study population and group sample sizes;
- Reported outcome quantitative and qualitative data;
- Statistical measures utilized in the study;
- Assessment tool used (e.g., Finnegan Neonatal Abstinence Scoring System [29,30] or the MOTHER NAS Scale [31]). Note, a higher score is indicative of increased NAS severity.

Three independent authors separately verified the data were accurately entered into the abstraction table (S.A., L.F., K.C.).

***Confidence in Cumulative Evidence***—The research team collated judgments about the underlying quality of evidence for the individual research articles selected using the Grading of Recommendations, Assessment, Development, and Evaluation (GRADE) approach [32]. The software tool GRADEpro (McMaster University, Hamilton, Canada) [33] was used to compile the data for evaluation. GRADEpro uses the following information to grade the strength of evidence from “HIGH to VERY LOW”: 1. Study design (experimental versus observational), 2. Factors that can decrease the quality (limitations in study design and/or execution, inconsistency of results, indirectness of evidence, imprecision of results, and publication bias), and 3. Factors that can increase the quality of evidence (large magnitude of effect, all plausible confounding may be working to reduce the demonstrated effect or increase the effect if no effect was observed, and dose–response gradient) [34]. A comprehensive listing of the variables and ratings, sorted by clinical trial or observational study, is included in the Supplementary Materials.

## 3. Results

**Figure 1:**
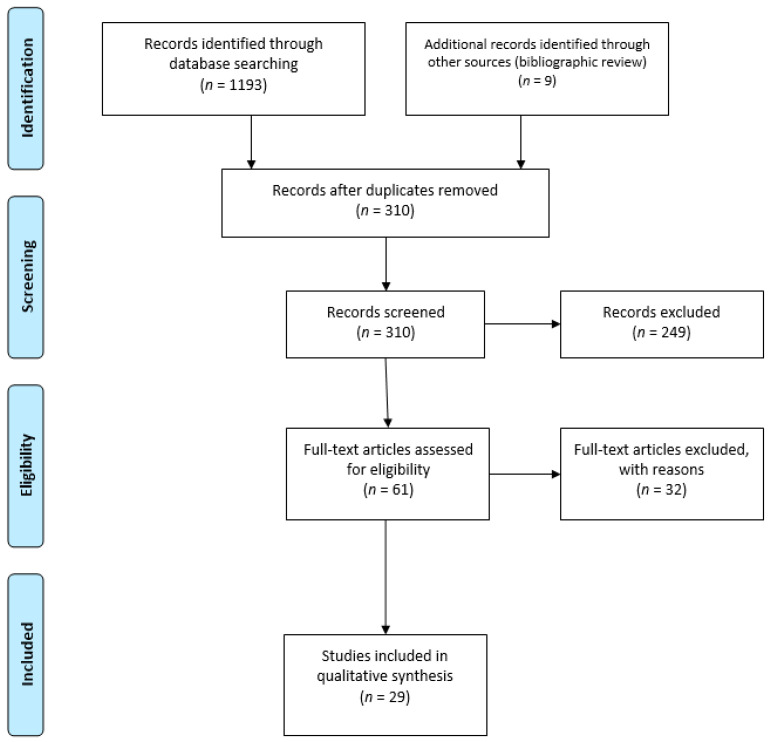
PRISMA flowchart of article identification and exclusion.

Out of 1202 papers identified through searches of Scopus and PubMed databases and bibliographic review, a total of 310 unique papers were screened after de-duplication and after an initial application of the inclusion and exclusion criteria to the study titles. An additional 249 of these 310 papers were excluded as they did not meet the eligibility criteria based on a review of the abstract. After a full-text review of the 61 remaining papers, an additional 32 articles were excluded for the following reasons: a total of 18 papers were excluded as there was no measurable outcome, one paper was excluded as it did not examine the combination of tobacco and opioid consumption, six papers were excluded as they only quantified tobacco and not opioid exposure, six papers were excluded as there was no information on an independent association between tobacco and NAS, and one paper was excluded as it was a review article. A total of 29 papers met the eligibility requirements for inclusion in this review (see Figure 1 and Table 1). Of this list of 29, nine were identified as clinical trials, three of which included data derived from the MOTHER study. Additional two clinical trials consisted of secondary data analysis of prior clinical trials (see Figure 1).

In this review of 29 articles, 11 papers examined only methadone exposure, four papers examined only buprenorphine exposure, nine papers simultaneously examined the combination of tobacco consumption and maternal pharmacotherapy treatment with either methadone or buprenorphine, two examined tobacco consumption in women who are pregnant with a history of documented polysubstance use including opioids, and three examined the impact of tobacco combined with prescription opioid use (see Table 1).

### 3.1. Methadone-Exposed Infants

Eleven papers examined whether there was an interaction between nicotine consumption and methadone use. Burns and Mattick (2007) found that mothers treated with methadone were more likely to have infants with NAS if they were consuming >10 cigarettes per day (CPD), were less than 25 years of age, were members of an indigenous population, and had no intramuscular analgesia during delivery. The infants of these mothers were significantly more likely to have a NAS diagnosis and to exhibit a longer duration of NAS (*p* < 0.001) [35]. Choo et al. (2004) reported that infants of mothers who were high-CPD smokers had significantly higher NAS peak scores (*p* = 0.014) and took longer to reach the peak score (*p* = 0.016) than infants of mothers with OUD who smoked less than 10 CPD, but they observed no difference in neonatal birth weight and head circumference for infants born to mothers who were high-CPD vs. <10 CPD smokers [36].

Two papers reported data suggestive of an impact on neonatal outcomes due to tobacco consumption. Seligman (2010) reported a nonsignificant increase in risk for NAS in infants of mothers being treated with methadone who also smoked (adjusted odds ratio = 2.2, 95% Confidence Interval (CI), 0.98–5.0). Note that the majority of the women in the study were smokers (*n* = 177 smokers vs. 18 nonsmokers) [37]. Ram et al. (2016) reported that women who successfully reduced their cigarette consumption during pregnancy exhibited a nonsignificant decrease in spontaneous abortions, higher infant birth weights, and a nonsignificant reduction in the number of infants being treated for NAS (*p*-values > 0.05 for all comparisons) [38]. The other papers [39,40,41,42,43,44] reported no neonatal outcomes that were significantly different in infants of mothers being treated with methadone who smoked vs. those who did not smoke (*p*-values > 0.05).

Jansson et al. (2010) reported that higher doses of cigarette use led to lower NAS scores on days 1 and 2 postpartum (correlation coefficient = −0.33, *p* < 0.01 and −0.28, *p* < 0.05, respectively) but had no theoretical explanation for this unexpected outcome [45]. Virtually all the women in the group ranged from 1 to 20 CPD.

### 3.2. Buprenorphine-Exposed Infants

Four papers assessed the interaction between nicotine consumption and buprenorphine use. Only one of these studies found a statistically significant impact on neonatal outcomes that could be attributed to tobacco consumption. O’Connor et al. (2011) reported that infants born to mothers undergoing buprenorphine treatment who smoked had significantly lower birth weights than infants born to mothers in buprenorphine treatment who did not smoke (*p* < 0.0136) [46]. Another study noted that there was a nonsignificant decrease in the number of infants exhibiting severe NAS when the mothers reported reduced cigarette smoking (mean maternal CPD for infants who had NAS was 17.5 compared with 9.44 CPD for mothers of infants without NAS, no statistical analysis was provided) [47]. The third paper [48] found no correlation between nicotine or its metabolites in the meconium and NAS expression or neonatal outcomes (statistical analysis not provided). The fourth paper [49] concluded that maternal cigarette exposure had no impact on neonatal outcomes, including NAS severity (*p* = 0.36). Note, all four papers that examined only buprenorphine had small sample sizes (*n* = 10 [48]; *n* = 15 [47]; *n* = 23 [46]; and *n* = 41 [49]), which made it much less likely that a statistical difference attributable to tobacco consumption could be detected.

### 3.3. Comparison of the Impact of Tobacco Consumption and Maternal Treatment with Either Methadone or Buprenorphine on Neonatal Outcomes

Nine studies directly compared whether tobacco consumption interacted with pharmacotherapy with either methadone or buprenorphine concerning neonatal outcomes for infants of mothers with OUD. Bakstad et al. (2009) reported the mean number of CPD correlated significantly with the length of treatment (LOT) for NAS for the methadone group (*p* < 0.023), but not the buprenorphine group (*p* = 0.7), although the women in the methadone treatment group smoked less than women in the buprenorphine group—methadone-treated women averaged nine CPD (range 1 to 20) while buprenorphine-treated women averaged 13 CPD (range 7 to 22) [50]. The peak NAS scores did not differ, and no statistical analysis was provided.

Chisolm et al. (2011) examined women with clinical depression in treatment for OUD as well as their non-depressed counterparts with OUD and saw a differential effect of CPD [51]. In women with no depression, each additional CPD increased the likelihood of NAS treatment in their infants by 12% (*p* = 0.02). In women with depression, each additional CPD did not impact the likelihood of NAS treatment. In a multivariate analysis, smoking was associated with low birth weight, defined as less than 2500 g (*p* < 0.01), and the need for NAS pharmacotherapy in infants exposed to either methadone or buprenorphine (*p* = 0.02).

Wachman et al. (2011) found no impact derived from smoking on the length of stay in the mothers treated with either buprenorphine or methadone, but it should be noted that many of the mothers in their studies were also taking benzodiazepines or selective serotonin reuptake inhibitors (SSRIs), which did increase the average length of stay to just under 23 days [52]. As such, the impact of nicotine may have been masked by psychiatric medications.

Kaltenbach et al. (2012) found that a greater number of cigarettes smoked 24 h before delivery predicted an increased need for pharmacological treatment of NAS (*p* = 0.03) and a need for a higher total dose of morphine (*p* = 0.05), whether or not the infant was exposed to methadone or buprenorphine [3]. 

Jones et al. (2013) reported that past-30-day daily average CPD significantly influenced a variety of neonatal outcome measures (e.g., the total amount of morphine to treat, length of stay, days medicated for NAS, and 5-min APGAR score, *p* < 0.01 for each) [4]. Although increased CPD resulted in more affected neonatal outcomes, the choice of maternal pharmacotherapy did not influence neonatal outcomes in women with OUD who also smoked.

Gibson et al. (2017) completed a linear regression analysis to determine if any potential confounding factors impacted the relationship between gestational age at birth and NAS severity. Their analysis revealed no effect of tobacco consumption on the length of treatment (LOT) for NAS with pharmacotherapy (*p* = 0.87 [53]).

A pilot study noted a nonsignificant increase in the Finnegan scores of neonates from mothers who consumed >10 CPD compared with mothers who consumed <10 CPD [54]. There was no discussion of whether this finding differed by maternal pharmacotherapy and no statistical analysis was provided.

Winklbaur et al. (2009) reported that an analysis of neonatal outcomes yielded significant differences dependent on CPD (note, in this study, low-CPD was defined as less than 10 CPD and high-CPD as greater than 20 CPD), with infants of low-CPD mothers in treatment for OUD showing higher birth weights, greater mean body lengths (*p* = 0.017), and greater head circumference (*p* = 0.054) than those born to high-CPD mothers [55]. Mean cumulative sums of the Finnegan scores were greater in neonates born to low-CPD mothers compared to high-CPD mothers (*p* = 0.030). The length of stay was also shorter for low-CPD mothers compared with high-CPD (*p* < 0.041).

Tolia et al. (2018), in a retrospective cohort study of 3364 eligible infants with NAS, compared the length of stay after antenatal exposure to either methadone or buprenorphine and reported no evidence of a correlation with tobacco consumption [56].

### 3.4. Polysubstance Exposure during Pregnancy

Two papers examined the impact of tobacco consumption on NAS and neonatal outcomes after exposure to multiple licit and illicit drugs. O’Donnell et al. (2009) conducted a retrospective cohort study with aboriginal and non-aboriginal women who are pregnant in Australia [57]. After examining 637,195 records, they reported that smoking increased the risk for NAS in both aboriginal (Odds Ratio (OR = 2.1) and non-aboriginal mothers (OR = 3.9). Subedi et al. (2017) investigated the levels of brain-derived neurotrophic factor (BDNF) in infants diagnosed with NAS due to opioid exposure and those who did not develop NAS after opioid exposure. They found that infants with NAS had higher levels of BDNF than those without NAS but that this finding was not influenced by tobacco consumption (*p* < 0.29) [58].

### 3.5. Prescription Opioid Use during Pregnancy

Patrick et al. (2015) conducted a multivariate analysis of prescription opioid use in women who are pregnant and found that the number of CPD was associated with a greater risk of developing NAS (*p* < 0.001 [59]). Desai et al. (2015) reported a similar outcome when they examined short-term and long-term prescription opioid use in women who are pregnant using a national Medicaid prescription database from 2000 to 2007 [60]. They confirmed that smoking elevates the risk of the infant exhibiting signs of NAS (risk factor 2.8−1.6×) compared to infants born to nonsmokers. The third paper by Shirel et al. (2016) examined the incidence of NAS in infants born to mothers who had sickle cell disease and who were using prescriptions for morphine to control the pain during pregnancy in a small number of cases (*n* = 30 pregnant) [61]. They reported that although doses of morphine greater than 200 mg/day were associated with an increased risk for NAS (13-fold), a history of tobacco use (in 6 of the 30 women) demonstrated no association with the presence or severity of the NAS (*p* = 0.59).

Additional data on the quality of the data provided in each paper are provided in the GRADEpro tables in the Supplemental Data file. In summary, for all categories of outcomes examined, the majority of the results were from observational studies where the certainty in the results was rated as low to moderate rather than clinical trials where the certainty was rated as high.

## 4. Discussion

The current scoping review provides evidence that prenatal tobacco exposure in women with OUD can have adverse effects on NAS severity and other birth outcomes, including low birth weight and reduced birth length. The review found no support for selecting a particular pharmacotherapy for OUD based on the interaction between pharmacotherapy and tobacco consumption. In practice, when selecting which type of pharmacotherapy is appropriate, the severity of the potential NAS should not be the criteria. Instead, the maternal history, including the severity of SUD, the duration of use and number of times in treatment, etc., should be used to make the decision, independent of whether the mother consumes tobacco or not. It should be noted that many studies found no differences in neonatal outcomes correlated with nicotine exposure. However, analyses of prescription data and the correlation of NAS severity and incidence provide some of the strongest evidence that opioid exposure in utero combined with tobacco consumption can exacerbate NAS severity. This points to the complexity of the issue, where there are still many unknown variables, including socioeconomic status, insurance status, and prenatal care, that can impact neonatal outcomes. Historically, tobacco use in women who are pregnant and have OUD or significant opioid exposure has been examined separately, with very little consideration of how simultaneous use of both substances could have an additive deleterious effect on neonatal outcomes. Larger, prospective, controlled studies need to be conducted to identify whether CPD alone can predict NAS severity and an impact on birth length and birth weight.

### 4.1. Study Limitations

Limitations of the review include the inability to extract consistent data from the studies due to the variability in rating scales and the heterogeneity of the populations. As such, a meta-analysis was not feasible. In addition, the use of self-report to document tobacco consumption and substance use may have introduced a number of reporting biases, such as social desirability and recall biases. Women who are pregnant with OUD typically have very high CPD rates (80% to 90% of the subjects in a study will be smoking daily during their pregnancies). Thus, studies require large sample sizes to have sufficient women with OUD who do not smoke in the comparison group to better understand and tease out the impact of nicotine exposure from the impact of opioid exposure. All of the buprenorphine-only studies reviewed had fewer than 30 mother–infant dyads, which does not provide a sufficient sample size to compare the difference in the additive effect of prenatal tobacco exposure between methadone versus buprenorphine-exposed infants. Additional studies need to be conducted with sufficient power to allow for statistical analysis. Quantifying tobacco exposure across the studies was not possible because few studies used formalized documentation of tobacco dependence, such as the Fagerstrom Test for Nicotine Dependence (FTND; [62]), or biochemical verification of tobacco use, such as cotinine levels in urine or saliva samples, but instead relied on informal self-report of CPD. In addition, a GRADEpro analysis (as presented in the supplemental table) indicated that the majority of papers identified were either of low or moderate certainty, rather than high certainty.

### 4.2. Recommendations for Tobacco Screening and Tobacco Cessation or Reduction for Women Who Are Pregnant with OUD

Most prenatal guidelines [63,64,65] recommend screening women who are pregnant with OUD for tobacco use while at the same time acknowledging that more research needs to be conducted to identify effective, evidence-based smoking cessation treatments for these women. While screening, brief interventions, and behavioral interventions have been recommended by the 2015 U.S. Preventive Services Task Force [66], the report states there is insufficient evidence regarding the balances and benefits of pharmacotherapy, such as nicotine replacement therapy and the use of electronic cigarettes for smoking cessation during pregnancy. SAMHSA released a toolkit consisting of three documents in September 2018 to support substance use treatment centers that are looking to offer tobacco cessation programs to their clients, consisting of a manual and quick guide for providers and a flyer for clients outlining the benefits of tobacco cessation during treatment for substance use disorders [67,68,69].

Additional research is necessary to identify the most successful interventions to help women who are pregnant with OUD to significantly reduce their nicotine consumption during pregnancy. A review on smoking cessation treatment for women who are pregnant with OUD [70] identified only three studies that examined the efficacy of smoking cessation programs for women with OUD [71,72,73]. Although brief intervention and skill training decreased smoking levels [72], only the study by Tuten et al. (2012) focused on smoking reduction among women who are pregnant in treatment with methadone for OUD and the use of financial incentives demonstrated a significant impact [72]. Active engagement in treatment to reduce smoking, as measured by numbers of collected urinalysis samples and mean days of treatment attendance, appeared to lead to a more successful smoking reduction, but even a 50% reduction in smoking was difficult to attain [38]. Recent publications indicate new studies are being planned to study interventions to REDUCE tobacco consumption in PPWOUD [74,75].

### 4.3. Future Directions

Given the limited coverage of the research literature on co-occurring opioid and tobacco use on NAS and neonatal outcomes, we compiled a list of research questions that may be useful in guiding future research (Table 2). There are numerous issues related to basic science that remain to be investigated, as well as issues about whether nicotine replacement therapy, electronic cigarettes, or smokeless tobacco use has an impact on neonatal outcomes in mothers with OUD. When researchers collected CPD on a daily or weekly basis to evaluate nicotine consumption as a continuous variable instead of a dichotomous one, it was more likely to yield useful information than a self-report on the maternal smoking impact on NAS [51]. Routinely using biomarkers such as cotinine to quantify exposure in both the mother and infant would further clarify some of the issues surrounding neonatal outcomes and nicotine exposure in infants that were also exposed to opioids. An observational or cohort study is needed to track differential additive effects of prenatal tobacco exposure between methadone- and buprenorphine-exposed infants. Based on the limited number of studies reported to date, it is clear further investigation is needed to examine the interactions among depression and/or anxiety [76] and tobacco consumption, opioid exposure, and NAS outcomes. Recent evidence showed that women who are pregnant and on opioid agonist therapy had a higher nicotine metabolite ratio compared to women who are pregnant but not with OUD [77]. Further research is necessary to examine a difference in the nicotine metabolite ratio between methadone and buprenorphine exposure.

More intervention research is needed to determine whether nicotine cessation programs are more effective in women who are pregnant with OUD treated with either methadone or buprenorphine and what is the best time to start the nicotine cessation program [78]. Implementation outcomes of sustained nicotine cessation programs for women who are pregnant with OUD are also needed to identify the facilitators and barriers as well as cost-effectiveness. Detailed suggested research questions are below.

## 5. Conclusions

There is still much work to be performed to explore the role of nicotine exposure in infants born to women with OUD and its effect on neonatal outcomes, including NAS. While the body of evidence suggests that nicotine exposure may exacerbate NAS in infants also exposed to opioids, additional studies with larger group sizes to compare by the status and type of opioid agonist therapy and an objective measure of tobacco exposure levels need to be undertaken. Being pregnant may provide the additional motivation for a woman to reduce or quit nicotine use, for both her health and that of her infant, and treatment providers can build on and harness such motivation by including smoking cessation programs for women who are pregnant who are also undergoing treatment for OUD.

## Figures and Tables

**Table 1:** Study design and outcomes reported.

Citation	Prenatal Opioid Exposure ^#^	Study Design	Study Sample Size	Neonatal Outcomes Reported *							
				GAD	BL	BW	CPD	PFS	TX	LOS	LOT
**Buprenorphine Exposure**											
Fischer et al., 2000	B	Clinical trial	15	x	x	x	x		x		x
Jansson et al., 2017	B	Prospective cohort study	41	x	x	x	x	x	x	x	x
Kacinko et al., 2008	B	Clinical trial	10	x	x	x	x	x	x	x	x
O’Connor et al., 2011	B	Retrospective cohort study	23			x		x			
**Methadone Exposure**											
Burns and Mattick 2007	M	Cross-sectional analysis	2941	x			x				
Choo et al., 2004	M	Clinical trial	29	x		x		x		x	x
Cleary et al., 2012	M	Prospective cohort study	114	x			x	x	x	x	
de Castro et al., 2011	M	Secondary analysis of randomized clinical trial	19	x			x				
Gray et al., 2010	M	Prospective cohort study	49	x	x	x		x	x	x	x
Jansson et al., 2007	M	Prospective cohort study	50	x	x	x	x	x	x		x
Jansson et al., 2010	M	Prospective cohort study	64		x	x				x	
Ram, Tuten and Chisolm 2016	M	Secondary analysis of randomized clinical trial	118			x	x		x		
Seligman et al., 2008	M	Retrospective cohort study	204				x				x
Seligman et al., 2010	M	Retrospective cohort study	386					x	x		
Velez et al., 2009	M	Prospective cohort study	77	x		x	x	x	x	x	
**Methadone OR Buprenorphine Exposure**											
Bakstad et al., 2009	M/B	Prospective cohort study	38	x	x	x	x	x	x		x
Chisolm et al., 2011	M/B	Clinical trial (MOTHER)	119	x		x	x		x		
Fischer et al., 2006	M/B	Clinical trial	14	x		x	x		x		x
Gibson et al., 2017	M/B	Retrospective cohort study	403			x			x	x	
Jones et al., 2013	M/B	Clinical trial (MOTHER)	131	x			x	x	x	x	
Kaltenbach et al., 2012	M/B	Clinical trial (MOTHER)	131			x	x	x	x		
Tolia et al., 2018	M/B	Retrospective cohort study	3364	x		x	x		x	x	
Wachman et al., 2011	M/B	Retrospective cohort study	273							x	
Winklbaur et al., 2009	M/B	Clinical trial	139		x	x	x	x	x	x	
**Polypharmacy Exposure**											
O’Donnell et al., 2009	Poly	Retrospective cohort study	637,195	x		x				x	
Subedi et al., 2017	Poly	Prospective cohort study	67	x		x		x	x	x	
**Prescription Opioid Exposure**											
Desai et al., 2015	RX	Retrospective cohort study	1,379,450				x			x	
Patrick et al., 2015	RX	Retrospective cohort study	112,029	x		x	x				
Shirel et al., 2016	RX	Retrospective cohort study	34	x		x		x	x		

Twenty-nine articles were identified for the scoping literature review. The reports are sorted by the source of prenatal exposure to opioids. X = outcome reported in the publication; ^#^ Neonatal Exposure: B = Buprenorphine only; M = Methadone only; M/B = Exposure to either methadone or buprenorphine; Poly = Polysubstance use; RX = Prescription opioids; * Neonatal Outcomes Reported: GAD = Gestational age at delivery; BL = Birth length; BW = Birth weight; CPD = Recorded cigarettes per day: PFS = Peak Finnegan score; Tx = Need for neonatal pharmacological treatment (Yes/No); LOS = Length of neonatal hospital stay; LOT = Length of treatment for NAS.

**Table 2:** List of potential future research investigations.

Basic Clinical Issues
Is there a differential influence of prenatal tobacco exposure on methadone- vs. buprenorphine-exposed neonatal outcomes?
What is the combined effect of prenatal nicotine and opioid exposure on the likelihood of sudden infant death syndrome?
If reduction is an attainable goal, what should be the maximum number of CPD? Would this number of CPD differ between women in treatment with methadone or buprenorphine? Note, whether the woman is encouraged to quit or reduce will largely depend on the CPD that the woman is smoking and the time in pregnancy when she comes to treatment. More CPD and later arrival to treatment will not permit a large decrease in CPD.
What would be quantitative differences in biochemical results of prenatal tobacco exposure by the status and type of opioid agonist therapy?
Is there an interaction between maternal depression, nicotine consumption, and NAS expression? Is there an interaction between medication for depression, nicotine consumption, and NAS expression?
What are the influences of combustible vs. smokeless tobacco, vapor or e-cigarettes, or nicotine replacement products on methadone- vs. buprenorphine-exposed neonatal outcomes?
Is there a difference in nicotine metabolite ratios between methadone- vs. buprenorphine-treated women who are pregnant and smokers?
**Treatment Issues**
What are the best treatment options (pharmacological and/or psychosocial) to treat prenatal smoking for women in treatment programs for OUD? What kind of interaction effect with methadone or buprenorphine treatment should be expected if pharmacotherapy was applied to treat prenatal nicotine use and birth outcomes?
Ideally, pre-conception would be an optimal time to start nicotine cessation to minimize fetal exposure. What is the optimal time to start a nicotine cessation program for a woman in treatment for OUD—at the induction onto methadone or buprenorphine? One or two months later? Pre-pregnancy? During pregnancy? Or early postpartum?
**Implementation Issues**
What are the critical components for an organization to adopt a prenatal nicotine cessation program?
What are the barriers and facilitators to implementing a nicotine cessation treatment program for women with OUD?
What is the cost-effectiveness in implementing and sustaining a nicotine cessation treatment program for women with OUD?

A variety of research gaps, sorted by whether they were basic science, treatment, or implementation issues, are listed.

## Data Availability

Not applicable.

## Supplementary Materials

The following GRADEpro tables are available online at https://www.mdpi.com/article/10.3390/ijerph181910215/s1, Table S1: Gestational Age at Delivery; Table S2: Neonatal Birth Length; Table S3: Neonatal Birth Weight; Table S4: Mean Cigarettes/per day; Table S5: Peak Finnegan Scores; Table S6: NAS Treatment; Table S7: Length of Stay; Table S8: Duration of NAS Treatment.
