# Supplementary material for: Neonatal Outcomes after Combined Opioid and Nicotine Exposure in Utero: A Scoping Review
Source: Int J Environ Res Public Health. 2021 Sep 28;18(19):10215. doi: 10.3390/ijerph181910215 (PMC8508043; doi:10.3390/ijerph181910215)
Supplement: Supplementary file 1 [file ijerph-18-10215-s001.zip › ijerph-1349633-supplementary.pdf]

Supplemental Materials-GRADEPro Analysis

Supplemental Materials: GRADEpro Analysis

Table S1. Gestational Age at Delivery

**Certainty assessment**							**Impact**	**Certainty**
**No. of studies**	**Study design**	**Risk of bias**	**Inconsistency**	**Indirectness**	**Imprecision**	**Other considerations**		
6	randomized trials	not serious	not serious	not serious	serious [a]	none	This review includes 6 clinical trial articles, with a total study population of 318 participants, examining the effect of nicotine on the opiate- maintained pregnant women and the gestational age at delivery in neonates. Neonates were exposed to buprenorphine (2 papers), methadone (1 paper), and either methadone or buprenorphine (3 papers).	⨁⨁⨁◯ MODERATE
13	observational studies	not serious	not serious	not serious	not serious [b]	none	This review includes 13 observational articles with a total study population of 756018 participants examining the role of nicotine on the opioid maintained pregnant women and the gestational age at delivery in neonates. Neonates were exposed to buprenorphine (1 paper), methadone (6 papers), either methadone or buprenorphine (2 papers), poly-substance use (2 papers) and prescription opioids (2 papers).	⨁⨁◯◯ LOW

CI: Confidence interval;

a. Results are imprecise as papers include relatively few patients and thus have a wide confidence interval (CI) around the estimate of the effect.

b. Large population size may lead to less confidence interval.

Table S2. Neonatal Birth Length

Certainty assessment							Impact	Certainty
No of studies	Study design	Risk of bias	Inconsistency	Indirectness	Imprecision	Other considerations		
3	randomized trials	not serious	not serious	not serious	serious [a]	none	This review includes 3 papers, with a total study population of 164 participants, examining the effect of nicotine on the opiate-maintained pregnant women and their neonates' birth length. The neonates were exposed to buprenorphine (2 papers) and to either methadone or buprenorphine (1 paper).	⊕⊕⊕◯ MODERATE
5	observational studies	not serious	not serious	not serious	not serious	none	This review includes 5 papers, with a total study population of 242 participants, examining the effect of nicotine on the opiate-maintained pregnant women and their neonates' birth length. The neonates were exposed to buprenorphine (1 paper), methadone (3 papers) and either methadone or buprenorphine (1 paper).	⊕⊕◯◯ LOW

a. Few patients have a wide confidence interval (CI) around the estimate of the effect.

Table S3. Neonatal Birth Weight

Certainty assessment							Impact	Certainty
**No of studies**	**Study design**	**Risk of bias**	**Inconsistency**	**Indirectness**	**Imprecision**	**Other considerations**		
8	randomized trials	not serious	not serious	not serious	serious [a]	none	This review includes 8 clinical trial papers, with a total study population of 579 participants, examining the effect of nicotine on the opioid-maintained pregnant women and the birth weight among neonates. Neonates were exposed to buprenorphine (2 papers), methadone (2 papers), and either methadone or buprenorphine (4 papers).	⊕⊕⊕◯ MODERATE
13	observational studies	not serious	not serious	not serious	not serious	none	This review includes 13 papers, with a total study population of 753434 participants, examining the effect of nicotine on the opioid-maintained pregnant women and the neonatal birth weight. Neonates were exposed to buprenorphine (2 papers), methadone (4 papers), either methadone or buprenorphine (3 papers), polysubstance use (2 papers), and prescription opioids (2 papers).	⊕⊕◯◯ LOW

a. Results are imprecise as papers include relatively few patients and thus have a wide confidence interval (CI) around the estimate of the effect.

Table S4. Mean Cigarettes/per day

Certainty assessment							Impact	Certainty
No. of studies	Study design	Risk of bias	Inconsistency	Indirectness	Imprecision	Other considerations		
8	randomized trials	not serious	not serious	not serious	not serious	none [a]	This review includes 8 clinical trial papers, with a total study population of 681 participants, examining the role of cigarettes per day (CPD) on the neonatal abstinence syndrome (NAS) and neonatal outcomes. Neonates were exposed to methadone (1 paper), buprenorphine (2 papers), and either methadone or buprenorphine (5 papers).	⨁⨁⨁⨁ HIGH
11	observational studies	not serious	not serious	not serious	not serious	none [b]	This review includes 11 observational studies, with a total study population of 1 498327 participants, examining the effect of nicotine on the NAS and neonatal outcomes. Neonates were exposed to buprenorphine (1 paper), methadone (6 papers), either methadone or buprenorphine (2 papers), and prescription opioids (2 papers).	⨁⨁◯◯ LOW

a. Sample size not published for treatment/control groups.
b. Publication bias around sample size is detected.

Table S5. Peak Finnegan Scores

Certainty assessment							Impact	Certainty
No of studies	Study design	Risk of bias	Inconsistency	Indirectness	Imprecision	Other considerations		
5	randomized trials	not serious	not serious	not serious	not serious	none	This review includes 5 clinical trial papers, with a total study population of 440 participants, examining the effect of nicotine on the opiate-maintained pregnant women and the peak Finnegan scores NAS neonates. The common outcome measured in these studies is peak Finnegan scores. The neonates were exposed to buprenorphine (1 paper), methadone (1 paper), and either methadone or buprenorphine (3 papers).	⊕⊕⊕⊕ HIGH
10	observational studies	not serious	not serious	not serious	serious [a]	All plausible residual confounding would reduce the demonstrated effect	This review includes 10 observational papers, with a total study population of 879 participants, examining the effect of nicotine on the opiate-maintained pregnant women and the peak Finnegan scores in neonatal abstinence syndrome (NAS) neonates. The common outcome measured in these studies is peak Finnegan scores. Neonates were exposed to buprenorphine (2 papers), methadone (5 papers), methadone or buprenorphine (1 paper), polysubstance use (1 paper), and prescription opioids (1 paper).	⊕⊕◯◯ LOW

a. Few patients have a wide confidence interval (CI) around the estimate of the effect.

Table S6. NAS Treatment

Certainty assessment							Impact	Certainty
**No. of studies**	**Study design**	**Risk of bias**	**Inconsistency**	**Indirectness**	**Imprecision**	**Other considerations**		
8	randomized trials	not serious	not serious	not serious	not serious	none	This review includes 8 papers, with a total study population of 677 participants examining the role of nicotine on the opioid maintained pregnant women and the treatment for neonatal abstinence syndrome (NAS). The common outcome measured in these studies is treatment required for infants with NAS. The neonates were exposed to buprenorphine (2 papers), methadone (1 paper) and either exposed to methadone or buprenorphine (5 papers).	⊕⊕⊕⊕ HIGH
11	observational studies	not serious	not serious	not serious	not serious	All plausible residual confounding would reduce the demonstrated effect	This review includes 11 papers, with a total study population of 4623 participants examining the role of nicotine on the opiate maintained pregnant women and the treatment for neonatal abstinence syndrome (NAS) in neonates. The common outcome measured in these studies is treatment required for infants with NAS. Neonates were exposed to buprenorphine (1 paper), methadone (5 papers), either methadone or buprenorphine (3 papers), poly substance use (1 paper) and prescription opioids (1 paper).	⊕⊕⊕◯ MODERATE

CI: Confidence interval

Table S7. Length of Stay

Certainty assessment							Impact	Certainty
No. of studies	Study design	Risk of bias	Inconsistency	Indirectness	Imprecision	Other considerations		
4	randomized trials	not serious	not serious	not serious	serious [a]	All plausible residual confounding would reduce the demonstrated effect	This review included 4 papers, with a total population size of 309, examining the effect of nicotine on the opiate-maintained pregnant women and their neonates. The common outcome measured in these studies is length of neonate's hospital stay. Neonates were exposed to buprenorphine (1 paper), methadone (1 paper), and either methadone or buprenorphine (2 papers).	⊕⊕⊕⊕ HIGH
11	observational studies	not serious	not serious	not serious	not serious	All plausible residual confounding would reduce the demonstrated effect	This review included 11 papers (with a total population size of 2021097), examining the effect of nicotine on the opiate maintained pregnant women and their neonates. The common outcome measured in these studies is length of neonate's hospital stay. Neonates were exposed to buprenorphine in 1 paper, methadone in 4 papers, either methadone or buprenorphine in other 3 papers, poly substance use in 2 papers and prescription opioids in 1 paper.	⊕⊕⊕◯ MODERATE

a. Results are imprecise as papers include relatively few patients and thus have a wide confidence interval (CI) around the estimate of the effect.

Table S8. Duration of NAS Treatment

Certainty assessment							Impact	Certainty
No of studies	Study design	Risk of bias	Inconsistency	Indirectness	Imprecision	Other considerations		
4	randomized trials	not serious	not serious	not serious	serious [a]	Strong association dose response gradient	This review includes 4 papers, with a total study population of 68 participants, examining the effect of nicotine on the opiate-maintained pregnant women and duration of neonatal abstinence syndrome (NAS) in neonates. The neonates were exposed to buprenorphine (2 papers), methadone (1 paper), and either methadone or buprenorphine (1 paper).	⊕⊕⊕⊕ HIGH
4	observational studies	not serious	not serious	not serious	serious [a]	All plausible residual confounding would reduce the demonstrated effect	This review includes 4 papers, with a total study population of 178 participants examining the effect of nicotine on the opiate maintained pregnant women and duration of NAS in neonates. The neonates were exposed to buprenorphine in one paper, methadone in 2 papers and either exposed to methadone or buprenorphine in other paper.	⊕⊕◯◯ LOW

a. Results are imprecise as papers include relatively few patients and thus have a wide confidence interval (CI) around the estimate of the effect.
